# Comparative Analysis of Oral Ondansetron, Metoclopramide, and Domperidone for Managing Vomiting in Children With Acute Gastroenteritis

**DOI:** 10.7759/cureus.47611

**Published:** 2023-10-24

**Authors:** Muazzam Ahmad, Anurag Rawat, Shireen Farrukh, Ihteshamul Haq, Arun Kumar Mandal, Asaf Syed, Muhammad Sajid

**Affiliations:** 1 Pharmacology, Khyber Medical College, Peshawar, PAK; 2 Interventional Cardiology, Himalayan Institute of Medical Sciences, Dehradun, IND; 3 Medicine, King Edward Medical University, Lahore, PAK; 4 Biotechnology and Genetic Engineering, Hazara University, Mansehra, PAK; 5 Medicine, Manipal College of Medical Sciences, Pokhara, NPL; 6 Internal Medicine, Ayub Teaching Hospital, Abbottabad, PAK; 7 Biotechnology and Genetic Engineering, International Islamic University, Islamabad, PAK

**Keywords:** domperidone, metoclopramide, ondansetron, antiemetic treatment, oral treatment, pediatric population, vomiting, acute gastroenteritis

## Abstract

Background

Acute gastroenteritis (AGE) is a major health concern in pediatric populations because of its associated vomiting, which worsens dehydration and the severity of illness.

Objective

The purpose of the research was to compare the relative effectiveness of oral ondansetron in treating AGE in children's vomiting when compared to oral domperidone and oral metoclopramide.

Methodology

A clinical investigation involving 120 pediatric patients diagnosed with AGE was conducted in Pakistan from November 2022 to April 2023 using a single-blind randomized design and convenience sampling. The participants received oral suspensions of ondansetron, metoclopramide, and domperidone, with doses of 0.15 mg/kg, 0.1-0.2 mg/kg, and 0.5 mg/kg, respectively, adjusted according to their body weight. The outcome in different groups was analyzed using the Statistical Package for the Social Sciences (SPSS) (version 20.0; IBM SPSS Statistics for Windows, Armonk, NY).

Results

At six hours, vomiting cessation rates were 80.0% for ondansetron (n=32), 72.5% for domperidone (n=29), and 67.5% for metoclopramide (n=27; p=0.29). By 24 hours, ondansetron exhibited significantly higher efficacy (92.5%; n=37) compared to domperidone (82.5%; n=33) and metoclopramide (77.5%; n=31; p=0.03). Adverse effects were minimal and comparable across groups.

Conclusion

Oral ondansetron demonstrated superior efficacy in managing AGE-related vomiting in children within 24 hours compared to metoclopramide and domperidone.

## Introduction

Acute gastroenteritis (AGE) is a significant global health concern, particularly among pediatric populations. It involves inflammation of the stomach and intestinal lining, and it is diagnosed based on clinical symptoms, primarily characterized by diarrhea and vomiting, often accompanied by abdominal pain, nausea, and occasionally fever [1,2]. Vomiting, a defining AGE symptom, not only causes dehydration but also increases the severity of the illness in children [3,4]. Antiemetic medications are now an essential part of therapy plans for combatting the negative effects of vomiting and the associated consequences. Because of their effectiveness in controlling vomiting episodes, ondansetron and domperidone have emerged as crucial choices among these treatment alternatives [5-8].

According to the United Nations Children's Fund (UNICEF), despite a 50% reduction in the global under-five mortality rate over the previous three decades, notable gaps remain between countries with low-to-middle income and those with high income. In the realm of preventable factors contributing to the death of children under five, infectious disorders such as diarrhea and pneumonia emerge as prominent contributors [9]. AGE is a significant cause of morbidity and mortality among children in underdeveloped countries. A global epidemiological analysis revealed 7.6 million under-five mortalities worldwide, with 64% linked to infectious causes. The primary infectious etiologies comprised pneumonia (14.1%), diarrhea (9.9%), and malaria (7.4%) [9,10]. However, there is still a glaring knowledge vacuum regarding the relative efficiency of various treatments, particularly when it comes to acute gastroenteritis in children in Pakistan [10].

This research aimed to clarify the relative effectiveness of oral ondansetron in treating acute gastroenteritis in children's vomiting when compared to oral domperidone and oral metoclopramide. Acute gastroenteritis episodes are common in Pakistan and tend to afflict children more than any other age group. Therefore, finding the best antiemetic treatment strategy is of utmost importance for raising the standard of care provided to these young patients.

The outcomes of this study have the potential to be used as markers to direct healthcare professionals' choices, resulting in carefully refined treatment plans for pediatric patients. This project aims to improve the quality of care provided to children suffering from vomiting caused by acute gastroenteritis by providing empirically supported insights into the most effective and well-tolerated therapies. This, in turn, holds up the possibility of bringing about improved clinical results, lower rates of morbidity, and an overall improvement in the well-being of this particularly vulnerable population.

## Materials and methods

Study design

A single-blind, randomized clinical investigation using a convenience sampling technique was conducted in Pakistan at the Khyber Teaching Hospital Peshawar and Ayub Teaching Hospital Abbottabad from November 2022 to April 2023. The study specifically targeted children diagnosed with acute gastroenteritis.

Participants

The study began with a preliminary participant sample of 160 children. However, because of cases of acute and chronic dehydration that necessitated hospitalization, a total of 25 children were forced to withdraw from the study. Furthermore, 15 people failed to comply with the follow-up procedures, resulting in their exclusion from the research. As a result, the research eventually included a cohort of 120 children who had been diagnosed with AGE.

Inclusion criteria

Children below the age of 10 years are eligible for inclusion in the study. To meet the criteria, they must have experienced a minimum of three episodes of non-bilious, non-bloody vomiting within a 24-hour period. Additionally, the presence of symptoms like bloating, abdominal pain, diarrhea, or sensations of uneasiness suggestive of AGE is considered, irrespective of whether they have a fever or not.

Exclusion criteria

Certain conditions preclude participation in the study. Individuals who have taken any anti-emetic medication within the four hours preceding enrollment or have a documented history of allergy to any anti-emetic medication are excluded. Severe diarrhea, defined as having more than one episode of defecation per hour, is also a criterion for exclusion [9]. Furthermore, individuals with preexisting medical conditions, including kidney or liver disorders, congenital heart abnormalities, neurological ailments, cancer, weakened immune systems, prior abdominal surgical procedures, or diabetes, should not be enrolled. Severe malnutrition, the presence of dysentery, an inability to tolerate oral feeding, or parental/guardian refusal to participate are additional factors that can result in exclusion from the study.

Group allocation and treatment protocol

An automated table generated by a computer was used to randomly assign the enrolled pediatric participants into three clearly defined groups. Children were administered oral ondansetron suspension in Group A (oral ondansetron) at a dosage of 0.15 mg/kg depending on body weight. For Group B (oral metoclopramide), metoclopramide was administered to the children, with a dosage ranging from 0.1 to 0.2 mg per kilogram of body weight. Conversely, Group C (oral domperidone) received oral domperidone suspension, with a dosage of 0.5 mg/kg relative to their body weight.

Medication administration, follow-up, and outcome measures

In the event of a child experiencing immediate vomiting after medication administration, a secondary dose of the designated antiemetic was administered within a 15-minute timeframe. After a span of 30 minutes following medication intake, children were permitted to consume fluids orally. Over a period of 6 hours post-medication, the children's condition was observed. Oral rehydration therapy (ORT) was initiated for those who had remained free from vomiting for a minimum of 45 minutes. Those in the three groups who successfully tolerated ORT were discharged with the prescribed antiemetic medication. Parents were encouraged to administer the prescribed antiemetic with the same medication, but only after 8 hours and if their children suffered sickness or continued vomiting after leaving the emergency department (ED). They were also instructed to return to the ED for a follow-up evaluation 24 hours after treatments ended.

Children who continued experiencing vomiting after the initial six-hour treatment period underwent a reassessment of their hydration status, and a determination was made regarding the necessity of their hospital admission. Those who showed signs of profound dehydration brought on by vomiting were taken to the hospital for proper care and later omitted from the research. To identify any recent events, parents were questioned on the incidence of vomiting incidences over the previous 24 hours at the follow-up appointment. In cases where vomiting had ceased after 24 hours, their antiemetic treatment was discontinued, and they were advised to continue with ORT. Reassessing the hydration status of individuals who were still throwing up led to a decision on whether to admit them. The major outcome was the proportion of children in every group who did not vomit within 24 hours following treatment and, thus, were taken as responders to oral antiemetics.

Statistical analysis

The outcome data were entered into SPSS (20.0) for analysis. The mean and standard deviation (SD) were calculated for continuous variables, such as age and the number of vomiting episodes. The primary outcome (the cessation of vomiting episodes) and categorical factors like gender were evaluated in terms of percentages and frequency. The chi-square test, which produces P values for statistically significant differences, was used to evaluate the frequency of the main outcome (termination of vomiting episodes) between each of the treatment groups, namely, oral ondansetron, oral metoclopramide, and oral domperidone. The significance threshold was set at p=0.05.

Ethical approval 

The study received ethical approval from the Institutional Research and Ethical Review Board of Khyber Medical College (IREB KMC).

## Results

The study's initial participant population was 160 kids. However, because of the acute and chronic dehydration that necessitated hospitalization, 25 kids had to be excluded, and a further fifteen kids failed for follow-up. As a result of these exclusions, the research ultimately comprised a cohort of 120 children diagnosed with acute gastroenteritis. Within this group of children, 51 (42.5%) were male, and 69 (57.5%) were female. These children were subjected to random allocation across three distinct groups, namely the oral ondansetron group, the oral metoclopramide group, and the oral domperidone group. Each of these groups comprised 40 kids. The participants in this research ranged in age from 4 to 55 months, with an average age of 29± 6 months. Table 1 contains information on these participants' characteristics at the start of the experiment.

**Table 1:** Comparative Analysis of Patient Characteristics in Ondansetron, Metoclopramide, and Domperidone Oral Treatment Groups for Acute Gastroenteritis-Related Symptoms SD: standard deviation n: number

Characteristics		Groups		
		Ondansetron	Metoclopramide	Domperidone
Age	Months (Mean±SD)	26±13	28±17	27±15
Gender	Male	19 (47.5%)	14 (35.0%)	18 (45.0%)
	Female	21 (52.5%)	26 (65.0%)	22 (55.5%)
Height	Centimeters (Mean±SD)	97.6±22.5	95.4±21.6	96.8±23.1
Body weight	Kilograms (Mean±SD)	12.9±5.6	13.2±5.2	13.6±5.9
Duration of diarrheal episodes	Hours (Mean±SD)	9.1±5.2	7.6±5.5	8.4±4.9
Number of diarrheal episodes	Last 24 hour (Mean±SD)	4±2	9±3	8±5
Duration of vomiting episodes	Hours (Mean±SD)	5.9±3.7	7.2±3.9	6.9±4.2
Number of vomiting episodes	Last 24 hours (Mean±SD)	8±5	5±4	6±4
No dehydration	n (%)	11 (27.5%)	16 (40.0%)	13 (32.5%)
Mild to moderate dehydration	n (%)	27 (67.5%)	22 (55.0%)	25 (62.5%)
Second dose of antiemetic	Required within 15 minutes	5 (12.5%)	9 (22.5%)	7 (17.5%)

After six hours, it was observed that 80% of children (n=32) in the ondansetron group experienced an improvement and cessation of vomiting episodes. Conversely, in the metoclopramide and domperidone groups, these figures were 67.5% (n=27) and 72.5% (n=29), respectively (Table 2). However, these differences lacked statistical significance (p>0.05).

**Table 2:** Comparison of Vomiting Outcomes Among Treatment Groups at 6 Hours: Oral Ondansetron, Oral Metoclopramide, and Oral Domperidone in Pediatric Patients with Acute Gastroenteritis n: number %: percentage p value <0.05 was considered significant

Treatment Groups	After a Six-Hour Period				P value
	Cessation of Vomiting		Persistent Vomiting		0.29
	n	%	n	%	
Oral Ondansetron	32	80.0	8	20.0	
Oral Metoclopramide	27	67.5	13	32.5	
Oral Domperidone	29	72.5	11	27.5	

At the 24-hour mark, the ondansetron group showed enhancement in 92.5% (n=37) of cases, as opposed to 77.5% (n=31) and 82.5% (n=33) in the metoclopramide and domperidone groups, respectively (Table 3). This difference was considered statistically significant, supporting the ondansetron-related outcomes (p=0.01).

**Table 3:** Comparative Evaluation of Vomiting Outcomes Among Treatment Groups at 24 Hours: Oral Ondansetron, Oral Metoclopramide, and Oral Domperidone in Pediatric Patients with Acute Gastroenteritis n: number %: percentage p value <0.05 was considered significant

Treatment Groups	After a 24-Hour Period				P value
	Cessation of Vomiting		Persistent Vomiting		0.03
	n	%	n	%	
Oral Ondansetron	37	92.5	3	7.5	
Oral Metoclopramide	31	77.5	9	22.5	
Oral Domperidone	33	82.5	7	17.5	

**Figure 1:**
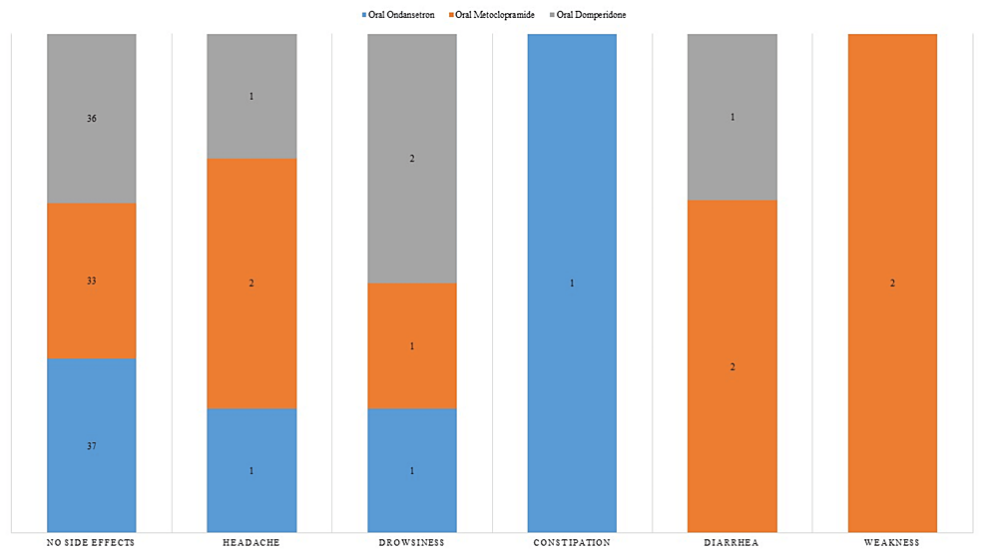
Comparative Analysis of Oral Ondansetron, Metoclopramide, and Domperidone: Incidence of Adverse Effects

The medications ondansetron, metoclopramide, and domperidone were relatively well-tolerated, with most users experiencing no side effects. Headache was infrequent, occurring in a few cases for each medication, while drowsiness and minor gastrointestinal issues like constipation or diarrhea were reported in limited incidence (Figure 1).

## Discussion

The management of AGE in children is a significant concern worldwide because of its potential to cause dehydration and exacerbate the severity of illness [10,11]. Vomiting, a common symptom of AGE, is particularly problematic as it leads to fluid loss and electrolyte imbalances. Antiemetic medications have become a vital component of treatment strategies to alleviate vomiting and its associated complications [12,13]. Ondansetron, domperidone, and metoclopramide have emerged as effective options for controlling vomiting in children; however, the comparative efficacy of these treatments, particularly in the context of acute gastroenteritis in Pakistani children, has not been well-studied [14,15]. This research addresses this gap by aiming to assess the effectiveness of oral ondansetron compared to oral domperidone and oral metoclopramide in managing vomiting associated with acute gastroenteritis in children in Pakistan.

In our study, we observed that within a six-hour timeframe, vomiting cessation was observed in 80.0% of children (n=32) who received ondansetron, as opposed to 72.5% and 67.5% of children in the domperidone (n=29) and metoclopramide groups (n=27), respectively (p=0.29). The initial rates of vomiting cessation after six hours did not demonstrate significant variations among treatment groups. Despite these trends, statistical analysis (p=0.29) did not establish a significant difference in vomiting cessation rates among the treatment groups within this initial timeframe. Nevertheless, by the 24-hour milestone, the superiority of ondansetron (n=37; 92.5%) compared to both the domperidone group (n=33; 82.5%) and the metoclopramide groups (n=31; 77.5%) became apparent. These results mirrored outcomes reported in previous research studies [16-20], strengthening the robustness of our findings and aligning with a growing body of evidence in favor of ondansetron's efficacy. These findings of current research are consistent with the results of other studies conducted in different populations. For example, a randomized controlled trial found that ondansetron was more effective than a placebo in reducing vomiting in children with AGE [21]. Another study found that ondansetron was more effective than metoclopramide in reducing vomiting and preventing dehydration in children with AGE [22].

The current study revealed that ondansetron demonstrates superior efficacy compared to domperidone and metoclopramide in alleviating vomiting in children with gastroenteritis, likely owing to its oral absorption and its role as a "serotonin 5-HT3 receptor antagonist." This mechanism involves suppressing the vomiting centers in the brain and inhibiting afferent depolarization of peripheral vagal nerves in the intestine, which can trigger emesis in gastroenteritis patients [12,23]. By reducing emesis, ondansetron may also promote oral fluid intake, potentially decreasing the requirement for hospitalization and intravenous rehydration [24]. Previous studies administered ondansetron, domperidone, and metoclopramide orally, with the oral disintegrating form of ondansetron offering a simpler and less-invasive administration method, particularly advantageous for vomiting children and those receiving outpatient care [9,16,25-30]. Furthermore, our study also delved into the safety profile of these antiemetic medications. Notably, the occurrence of adverse effects from the medications was minimal and comparable among the groups. This underlines the favorable overall tolerability of ondansetron, domperidone, and metoclopramide alike, offering a reassuring aspect for their clinical use.

The limitations of this study include a relatively small sample size, a short 24-hour observation period, the single-blind design, geographic specificity to Pakistan, fixed doses adjusted by body weight, and a focus solely on vomiting cessation rates without considering other clinical outcomes or potential confounding factors related to ondansetron, domperidone, and metoclopramide. Conducting a short-term and small-sized trial study for evaluating treatments in pediatric acute gastroenteritis offers advantages in terms of cost-efficiency, quicker results, reduced participant burden, and ethical considerations, especially in contexts like Pakistan where conducting long-term studies can be challenging because of resource limitations and other logistical constraints. These studies can provide initial insights into treatment efficacy and are particularly useful when resources and time are limited. However, they may have limitations in detecting rare side effects and assessing long-term outcomes, making them valuable preliminary investigations.

## Conclusions

This study provides valuable insights into the management of vomiting associated with AGE in children. By analyzing how well oral ondansetron, oral metoclopramide, and oral domperidone worked, the study shows that ondansetron may help improve clinical results in children. The findings contribute to evidence-based decision-making for healthcare professionals in Pakistan and, potentially, in similar settings globally. However, further research and broader studies are warranted to validate and generalize these findings, considering variations in patient populations, clinical conditions, and regional factors.
